# The oral‒gut axis in periodontal disease: a systematic review of the associated human evidence of its mechanisms and implications for patient care

**DOI:** 10.1080/20002297.2026.2705699

**Published:** 2026-07-22

**Authors:** Edward Coote, Nitesh Patel, Rajesh Vijayanarayanan

**Affiliations:** a 21D Clinical, Warrington, UK

**Keywords:** Periodontitis, gut microbiome, oral–gut axis, intestinal permeability, systemic inflammation, drug metabolism

## Abstract

**Background:**

Periodontal diseases may influence systemic health through the oral-gut axis, with three mechanistic pathways proposed. Periodontitis-associated gut dysbiosis has been repeatedly observed and may plausibly extend to effects on drug metabolism, yet the literature on periodontitis-associated dysbiosis and that on its potential effects on medicine pharmacokinetics have not previsouly been bridged.

**Objective:**

To synthesise the available human evidence for each of the three pathways with a distinct focus on the interventional evidence, and to position the evidence to its clinical context alongside downstream outcomes and the implications for patients and clinicians. Our secondary aim was to connect the dysbiosis and pharmacokinetic literatures in a hypothesis-generating section.

**Design:**

This systematic review was registered on PROSPERO. PubMed and Scopus were searched and eligibility restricted to human studies.

**Results:**

A total of 76 studies were included following full-text assessment by two independent reviewers. Across the three pathways, the human evidence was placed in clinical context with its downstream outcomes. Direct evidence that periodontitis modeifies drug response in humans was not identified, however, we were able to provide a hypothesis-generating section that identifies four drug classes for which a periodontitis-mediated modification of drug response would be most clinically relevant.

**Conclusions:**

The oral-gut axis is a biologically plausible but, currently, under-evidenced route from periodontal disease to systemic and pharmacological outcomes. Future reserach should prioritise prospective comparative pharmacokinetic studies in periodontitis-affected popualtions and field-wide standardisation of methodology across oral-gut microbiome research.

## Introduction

Globally, projections indicate that the prevalence of periodontal diseases will increase by 39.7% by 2040, representing significant disease growth [1]. In 2021, severe periodontitis affected over 1 billion people globally, giving an age-standardised prevalence of 12.5% [2]. Oral diseases, including periodontal disease, are now more commonly seen as part of a wider systemic inflammatory network [3]. Large-scale observational studies have consistently demonstrated significant associations between periodontal diseases and cardiovascular diseases, diabetes, rheumatoid arthritis, and more recently, cognitive decline [4–7].

The oral–gut axis has emerged as an aetiological mechanism behind the association between periodontal disease and systemic diseases. Studies have demonstrated that oral bacteria may prompt pathological changes associated with various diseases developing through gut microbiota dysbiosis [8]. Key pathogens involved in this process include Porphyromonas gingivalis, Fusobacterium nucleatum, Aggregatibacter actinomycetemcomitans, Tannerella forsythia and Treponema denticola [9,10]. Certain oral microbes spread through the body through three potential pathways: (1) translocation, with oral bacteria swallowed in saliva reaching the gut; (2) immune-mediated systemic signalling, with the release of cytokines worsening gut inflammation and altering gut barrier function; and (3) oral bacteria producing metabolites that influence the gut microbial composition [11–13]. Despite this growing mechanistic understanding, translational evidence for patients remains limited.

The oral–gut axis has been reviewed several times in recent years. These syntheses are valuable but share three features that limit their use for clinicians: they are narrative in methodology; they combine human, animal, and *in vitro* evidence without distinguishing the strength of each; and they conclude at the level of disease association [8,14,15]. None is restricted to human evidence, and none extends the axis to its consequences for medication response. We do not re-summarise the established translocation and immune-signalling associations; instead, we take those as a foundation and concentrate primary-study synthesis on the under-examined links, such as the effects of periodontal therapy on gut and metabolite outcomes, and the previously unbridged intersection between periodontal status and drug metabolism.

We identified this gap as clinically important, as patients presenting with moderate-to-severe periodontitis commonly suffer from systemic diseases and carry a higher disease burden than the general population [16,17]. The human evidence on the interaction between periodontal therapy and the oral–gut axis, therefore, warrants examination. As an extension of this, we consider it important to examine the medication effect and what is currently known about how oral–gut dysbiosis affects medications [18]. Rather than resting on the known oral–gut associations that other reviews have already discussed, we take a patient-clinician perspective on the consequences of treating individuals with moderate-to-severe periodontitis for oral–gut dysbiosis [8], and we integrate a section on how oral–gut dysbiosis can alter the pharmacokinetics of medications [19].

## Methodology

**Figure 1.**
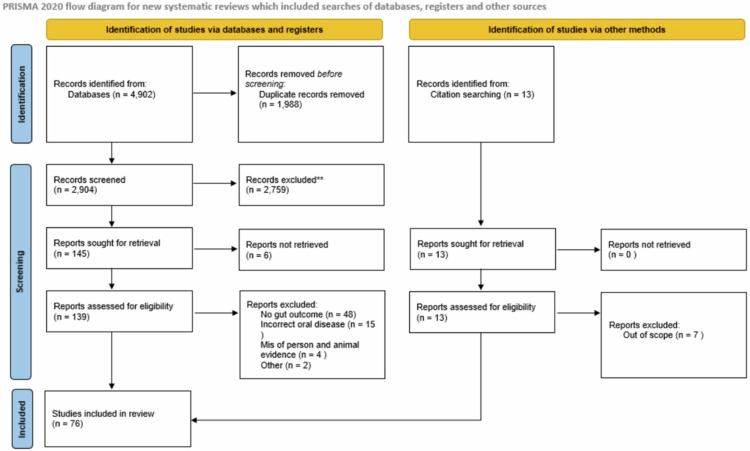
PRISMA 2020 flow diagram showing identification, screening, eligibility and inclusion of studies.

This systematic review was conducted and reported in accordance with the PRISMA 2020 statement and was registered on PROSPERO (CRD420261437988). The evidence was synthesised narratively in line with the Synthesis Without Meta-analysis (SWiM) guidelines. PubMed/MEDLINE and Scopus were searched from inception to June 2026. The full search strings for each database are listed in Supplementary Material Table S1. Titles and abstracts, then full texts, were screened against the eligibility criteria by EC and NP. Full-text disagreements over inclusion were discussed between all three authors. Reasons for exclusion at the full-text stage are summarised in the PRISMA 2020 flow diagram (Figure 1).

Three pre-specified questions were addressed: (1) whether periodontal disease is associated with altered gut microbiota composition; (2) whether periodontal therapy alters gut microbial, systemic inflammatory, or microbial-metabolite outcomes; and (3) whether periodontal status modifies the pharmacokinetics or therapeutic response of orally administered drugs. The PECO for each question is defined in Table 1.

**Table 1.** PECO framework for systematic search.

Criterion	Inclusion	Exclusion
**Population (all three questions)**	Adults (≥18 years) with a clinical diagnosis of periodontitis, defined by any recognised system (2017 World Workshop staging/grading; CDC–AAP case definitions; or explicit clinical attachment loss/probing pocket depth criteria), with the case definition recorded per study. Moderate-to-severe periodontitis (Stage III– IV) eligible throughout.	Paediatric or adolescent-only populations; fully edentulous participants; gingivitis or periimplant disease in the absence of a periodontitis group; populations in which periodontitis status cannot be ascertained.
**Exposure/Intervention**	**Q1 (exposure):** presence of periodontitis. **Q2 (intervention):** periodontal therapy—non-surgical (scaling and root planing/subgingival instrumentation) or surgical, with or without adjuncts (systemic or local antimicrobials, probiotics). **Q3 (exposure):** periodontal status as a candidate modifier of drug handling.	Oral interventions not directed at periodontitis (e.g. caries management, orthodontics); pharmacological studies in which periodontal status is neither measured nor reported.
**Comparator**	**Q1/Q3:** periodontally healthy individuals (or, in within-cohort designs, participants without periodontitis).	Studies without any comparator or baseline measurement.
	**Q2:** no treatment, delayed/deferred treatment, or the participant’s own pretreatment baseline (single-arm before–after designs).	
**Outcomes**	**Primary:** gut microbiota composition (alpha/beta diversity, Firmicutes/Bacteroidetes ratio, relative abundance of named taxa). **Secondary:** systemic inflammatory markers (CRP, IL-6, TNF-*α*, circulating LPS/endotoxin); microbial metabolites (SCFAs, TMAO, salivary/faecal metabolomic signatures). **Q3:** any pharmacokinetic parameter (e.g. AUC, Cmax) or measure of therapeutic response, or drug/metabolite levels.	No eligible outcome reported. Downstream clinical endpoints (IBD, colorectal cancer, cardiovascular events, cognitive decline, type 2 diabetes control) are not primary search targets; these associations are synthesised at the level of existing systematic reviews as umbrella-level context, not re-reviewed from primary studies.

Eligible studies were: observational (cross-sectional, case‒control, cohort) and randomised and non-randomised interventional studies, including single-arm designs. Reference lists of relevant existing reviews were handsearched to ensure the complete inclusion of all relevant evidence. Animal, *in vitro*, ex vivo, and in silico studies, reviews, editorials, commentaries, conference abstracts and studies lacking a comparator or an eligible outcome were excluded. Only studies in English were included.

Systemic inflammatory markers (CRP, IL-6, TNF-*α*, and LPS/endotoxin) were pre-specified as eligible outcomes because immune-mediated systemic signalling is one of the three oral–gut pathways. Periodontal inflammation increases the levels of circulating cytokines and endotoxins that act on the gut barrier, and these markers are the principal measurable endpoints of that pathway. Inflammatory-marker studies were eligible, but findings are synthesised specifically for their bearing on gut–barrier signalling, with the broader periodontitis–systemic-inflammation literature used as context. Gingival crevicular fluid short-chain fatty acids were included as metabolite pathway outcomes because they are direct products of subgingival microbial metabolism and represent the local, in-pocket origin of the metabolite signalling that the oral–gut axis proposes operates bidirectionally.

The risk of bias was assessed using RoB 2 for randomised trials, ROBINS-I for non-randomised interventional studies, the Newcastle‒Ottawa Scale for observational cohort and case‒control studies and the JBI critical appraisal tool for cross-sectional studies [20–23]. ROBINS-I was also used for Mendelian randomisation. Owing to large heterogeneity in periodontitis case definitions, biological sampling sites (saliva, stool, and blood), measurement platforms (16S rRNA amplicon versus shotgun metagenomic sequencing versus targeted metabolomics), and outcome metrics, evidence was not formally graded with GRADE. Publication bias was not formally assessed, as funnel plots are inapplicable without meta-analysis. Mechanistic and pathway-level literature is presented as a narrative context and supporting evidence that was not subject to the systematic search.

## Results

The database searches identified 4,902 records across PubMed/MEDLINE and Scopus, with 13 further records identified through hand-searching reference lists and trial registries. After 1,998 duplicates were removed, 2,904 records were screened by title and abstract, of which 2,759 were excluded. A total of 158 full-text articles were sought for retrieval, 6 of which could not be accessed. There were 152 reports assessed for eligibility, 76 were excluded (48 for no eligible outcome, 15 wrong population or disease entity, 4 for not human, 7 out of scope, 2 for other), leaving 76 studies included in the review.

Twenty-eight studies addressed the association between periodontitis and the gut microbiota composition, 47 addressed the effect of periodontal therapy on gut microbial, inflammatory or metabolite outcomes, and 4 addressed the effect of periodontal status on drug pharmacokinetics or response. The 36 included studies were cross-sectional, 2 case‒control, 11 cohort, 2 randomised controlled trials, 6 Mendelian randomisation and 19 non-randomised or single-arm interventional studies.

Cross-sectional studies appraised with the JBI checklist (*n* = 38) were largely judged to meet the quality criteria. Non-randomised interventional studies appraised with ROBINS-I (*n* = 18) were predominantly at serious risk of bias, mainly due to confounding. The 6 Mendelian randomisation studies, appraised with a ROBINS-I framework adapted for Mendelian randomisation, were predominantly at moderate risk of bias. Cohort studies appraised with the Newcastle‒Ottawa Scale (*n* = 11) and 2 case‒control studies appraised with the same tool were predominantly of fair quality, with inadequate follow-up duration and outcome ascertainment relative to baseline being the most common concerns. One quasi-experimental study was appraised with the JBI quasi-experimental checklist. The two randomised controlled trials were both rated as having a low risk of bias; however, the fact that there were only two randomised controlled trials in total is a cause of concern for the evidence base. A full summary of all risk-of-bias analyses can be found in the Supplementary Material (Table S2).

## Discussion

The oral microbiome can be linked to the gut through the discussed three pathways. These processes likely operate simultaneously in an individual with periodontitis, and the complexity of human biology makes it difficult to isolate the causal relationship and effects of single pathways. We discuss the primary evidence of each pathway individually owing to its clinical relevance and pedagogical utility when analysing the oral–gut axis and considering patient and treatment implications. A healthy microbiome represents a functionally stable microbial ecosystem that supports host homeostasis, limits pathobiont expansion, and maintains balanced immune and metabolic signalling. Rebalancing describes movements away from dysbiosis and towards functional stability.

### Pathway 1 – direct bacterial translocation

#### Observational evidence

Studies using paired saliva-stool samples have observed that the translocation of oral bacteria into the gut commonly occurs in community-dwelling adults, and that ageing and dental plaque build-up are positively associated with increased numbers of oral microbes in the gut [24,25]. In observational studies that have specifically recruited individuals with moderate-to-severe periodontitis, this translocation trend intensifies. Using 16S amplicon sequencing of paired saliva and faecal samples, researchers observed greater leakage of oral bacteria into the gut in individuals with periodontitis than in oral-healthy controls, with parallel shifts in faecal community composition, including increases in Bacteroides, Faecalibacterium, Fusobacterium and Lachnospiraceae [26,27]. Reductions in faecal alpha diversity from a healthy periodontal state to periodontitis have also been observed [28,29]. Individuals with periodontitis have also shown enrichment of Desulfovibrio fairfieldensis, Erysipelothrix tonsillarum, and Peptostreptococcus anaerobius in the faeces, with dysbiosis extending to clinically healthy oral sites as well [30]. Using a high number of teeth as an indicator of periodontal health, cross-sectional analysis observed few associations between oral and gut bacteria and metabolites in either elderly or healthy subjects, which indicates few detectable oral–gut associations in healthy subjects [31]. The significant differences between the saliva-sourced microbes detected in faecal samples of individuals with severe periodontitis and those of periodontally healthy participants support the idea that periodontitis may promote gut microbiota dysbiosis through a translocation influx of salivary microbes through the gastrointestinal tract [32]. Research has extended this connection to determine that parental periodontitis, together with the distinct associated faecal microbiota profile associated with it, appears to influence the faecal microbiome and systemic markers of their children. [33].

The majority of studies use 16S rRNA sequencing, which has several limitations. One constraint is that targeting only one or a few hypervariable regions typically restricts identification to the genus level and resolves species only unreliably. Relative to shotgun metagenomics, 16S sequencing has lower resolution and sensitivity, detecting substantially fewer genera and capturing only broad community shifts [34,35]. That being said, a large multi-cohort shotgun analysis has questioned the extent of oral–gut translocation in healthy adults [36].

Metagenomic analysis has suggested that translocation may extend beyond the gut lumen. In individuals with severe obesity and periodontitis, bacterial DNA has been found in visceral adipose tissue, predominantly from gut-associated genera and species alongside some oral-associated taxa [37].

#### Interventional evidence

Periodontal therapy has demonstrated the ability to shift the gut microbiota of individuals with periodontitis towards a composition similar to that of periodontally healthy individuals [27,38]. Non-surgical periodontal therapy (NSPT) reduces the ratio of Firmicutes/Bacteroidetes in the gut, an indicator of improvement in the gut microbiome [32]. In the mouth, periodontal therapy reduces *P. gingivalis* and other key pathogens, and commensal taxa such as Veillonella parvula, Streptococcus mitis, and Streptococcus oralis re-emerge as part of a healthy oral microbiome [39]. A significant decrease in faecal F. nucleatum levels has also been demonstrated in patients with colorectal tumours after periodontal therapy; however, its ability to slow colorectal tumour growth or prevent development has not been tested [40].

Overall, the evidence base contains small samples and short follow-up periods, which limit the ability to determine whether these changes persist long-term after treatment. Interventional evidence, especially within the translocation pathway, remains thin because interventions primarily target systemic inflammatory diseases rather than translocation-specific endpoints [41,42].

### Pathway 2 – Immune-mediated systemic signalling observational evidence

The available observational evidence suggests that individuals with periodontitis have increased systemic levels of inflammatory markers such as CRP, IL-6, TNF-*α* and LPS, which have been implicated in gut barrier dysfunction and further systemic inflammation [43–45]. Studies pairing salivary cytokine measurement with faecal microbiome sequencing have linked inflammation sourced from the oral cavity to gut dysbiosis. Salivary IL-6 and inflammatory chemokine levels have been shown to positively correlate with the abundance of recognised oral pathobionts, while simultaneously negatively correlating with commensal taxa such as Rothia aeria and Haemophilus parainfluenzae [46]. Further, known pathogenic oral taxa, including *P. endodontalis* and *Prevotella* have been detected in the gut, which are linked to inflammatory diseases [47].

Cross-sectional data cannot firmly establish causal directionality alone, but the evidence indicates a cyclical, bidirectional relationship in which periodontal inflammation contributes to systemic immune activation and gut dysbiosis, which may in turn lead to further periodontal inflammation. Mendelian randomisation studies can support a causal component to this bidirectional relationship. Two-sample Mendelian randomisation studies have reported causal effects of specific gut microbial taxa on periodontitis risk [48,49]. Bidirectional analyses have indicated these effects in both directions [50,51]. One study extends this from the oral microbiome and through the host metabolism and inflammation to periodontitis [52].

Observational evidence linking inflammatory biomarkers, periodontal diseases and gut health is limited because oral health markers and stool samples are not routinely collected at the population scale, for example, in the UK Biobank. This means that the majority of evidence on immune biomarkers comes from individual clinics with small sample sizes and a minimal follow-up period.

In a large Finnish cohort, exposure to periodontal pathogens and elevated serum antibody levels induced inflammation and led to an increased risk of cardiovascular diseases [53]. The previously mentioned systemic inflammatory markers have been hypothesised as the reason for this increased risk. Different observational cohort studies have extended this link to other hard disease endpoints, such as strokes, vascular damage, psoriasis and sclerosis, revealing a bidirectional relationship between periodontitis and these diseases [54–57]. Periodontitis has also been associated with endotoxemia, with lipopolysaccharides suggested as the molecular mediator between periodontitis and inflammatory diseases [58–60]. The effects of residual confounding by shared risk factors must be considered when evaluating this observational evidence. Marker studies measure association, and not mechanism, which introduces reverse causation.

#### Interventional evidence

Interventional efforts that target systemic inflammation tend not to integrate the gut angle or report improvements in gut health. Direct interventional evidence on gut outcomes through controlling inflammation is limited. However, interventional evidence under this pathway is more disease-endpoint focused, which is highly relevant for the patient-clinician applications. Here, the evidence provides important context, but assumes a causal leap to improve gut health and its bidirectional, consequential improvements in reducing systemic inflammation. One study with only 90 days of follow-up demonstrated that NSPT in a chronic periodontitis group reduced IL-6 by 12.02 pg/mL, but the level of IL-6 was the only inflammatory marker that demonstrated a statistically significant reduction in the chronic periodontitis group [61]. It also found a directly proportional correlation between changes in probing depth, clinical attachment level and CRP levels; however, the study is limited by a short follow-up time, so long-term changes cannot be assessed. One study has demonstrated that probiotics can be used to manage periodontitis alongside NSPT through balancing the oral and gut microbiome, via their ability to increase anti-inflammatory cytokines, such as IL-10, and reduce pro-inflammatory cytokines [62,63]. Inflammatory markers without adjuncts have also been decreased through non-surgical periodontal therapy, with one study demonstrating glycaemic control via inflammation reduction and a reduction in cardiovascular risk [64–67].

### Pathway 3 – Metabolite-mediated effects

#### Observational evidence

TMAO is derived from gut-bacterial trimethylamine via hepatic oxidation, and elevated levels have been detected in individuals with periodontitis [68,69]. TMAO is gut-derived, so its elevation implies signalling in the oral-to-gut direction. One observational study has furthered this to associate circulating TMAO levels with vascular endothelial dysfunction, and another associated carotid atherosclerosis in a northeast Chinese cohort; however, in both cases, causation could not be confirmed [70,71]. A pilot study helped to distinguish consistent metabolite signatures in individuals with periodontitis, which has been extended to other metabolite classes, such as the elevation of polyamines, ornithine decarboxylase and oxidative-stress markers across gingivitis and successive periodontitis stages, alongside a rising Streptococcus abundance, proposed together as a severity marker [72,73]. Observational evidence predominately works towards diagnostic biomarkers for periodontal disease. One study stratified patients into two subgroups distinguished by their metabolite profiles, one showing higher short-chain fatty acid concentrations and a greater abundance of pathogenic taxa, supporting multi-omic metabolite profiling as a route to subtyping and treatment prediction of periodontal disease [74]. Salivary metabolomic studies have consistently identified metabolite signatures that distinguish individuals with periodontitis from healthy controls [75,76].

Periodontitis-associated metabolite signatures are detectable in the gut as well as the mouth. Combined oral and faecal analyses have found elevated pro-inflammatory metabolites, including succinate and trimethylamine, along with reduced SCFAs and amino acid levels, in both saliva and stool [77–79].

The human evidence for metabolite-mediated effects is thinner than that for the previous two pathways and is predominantly cross-sectional. The consistent identification of altered metabolite signatures in the saliva and stool samples of individuals with periodontitis supports further research into the metabolite-mediated angle of the oral‒gut axis.

#### Interventional evidence

Here, studies have measured the metabolites found before and after periodontal treatment using metabolic analysis, although the link to tangible changes in general health due to improvements in the oral‒gut axis is limited. One study found that NSPT resulted in a significant improvement in the metabolite profile of periodontitis patients, although it only resulted in a 3-month follow-up [80]. Non-surgical therapy reduces gingival crevicular fluid acetic, propionic and butyric acid concentrations, and crevicular volatile fatty acids track subgingival recolonisation before and after treatment [81,82]. Similar results were observed in a slightly larger sample size, which described nonsurgical periodontal treatment as improving the metabolic profiles of individuals with chronic periodontitis, but with post-treatment metabolic profiles still being distinct from those of healthy subjects [83]. Furthermore, a multiomics study found the salivary and faecal metabolome only partially reverted toward health after therapy, especially in saliva, rather than in stool [39]. Metabolic and microbial normalisation after therapy has mixed evidence, with the oral microbiome and metabolome dysbiosis persisting after treatment in an observed cohort [84].

### Clinical context and what this means for patients

Inflammatory bowel disease (IBD) connects most directly to the translocation pathway, with recent evidence demonstrating that bacteria that normally reside in the oral cavity appear in the luminal contents and mucosal tissues of the intestines in individuals with IBD [85,86]. Oral pathobionts travel from the oral cavity through the gastrointestinal tract and persist in the intestinal tract. Evidence from matched saliva and faecal samples from IBD patients with and without periodontitis reinforces this clinical relevance, demonstrating that the gut microbial profile of IBD patients more closely resembles their own oral microbiota than that of healthy individuals [86]. Current evidence positions *P. gingivalis* as the most likely translocator, given its ability to tolerate acidic environments through protective salivary components [87]. One study reported that the abundance of Porphyromonadaceae in faecal samples from patients with Crohn’s disease (a common manifestation of IBD) was significantly higher than that in the control group [88]. In addition, ulcerative colitis (another common manifestation of IBD) has been observed to occur more frequently in patients with periodontitis in large cohort studies [89,90]. Periodontal therapy that targets a rebalancing of the oral‒gut axis biomes may offer benefits extending beyond the oral cavity, through the removal of harmful translocating organisms. In this way, better oral hygiene acts as a barrier to gut exposure and may reduce the bacterial load reaching the gut, with potential IBD risk reduction. Periodontal care should form part of integrated disease management alongside gastroenterological efforts, rather than remaining a separate dental matter. The more inflamed and deeper the periodontal pockets, the greater the amount of harmful bacteria load patients swallow, owing to higher concentrations of *P. gingivalis* and *T. forsythia* [91]. The current evidence suggests positioning periodontal assessment as an adjunctive to IBD care, and clinicians may place oral hygiene as a moderate mediator that acts on the gut. Oral-stool profiling showed that the gut microbiota of IBD patients resembles their own oral biome more than that of periodontally healthy individuals, and that periodontal disease tracks with IBD activity [92,93]. Although no trial has yet shown that treating periodontitis improves IBD, the bidirectional nature of the relationship makes it relevant to clinicians identifying oral hygiene as a low-risk upstream target pending confirmatory interventional evidence.

Individuals with IBD also form a high-risk group for colon cancer (CRC) development, indicating that CRC is a severe potential downstream outcome associated with gut neglect and dysbiosis. The strongest mechanism explaining this phenomenon involves the role of Fusobacterium nucleatum (which originates in the oral cavity), which appears to be enriched in colorectal tumours [94,95]. Large prospective cohort studies have associated periodontitis with higher CRC incidence [96,97]. Translocation is one proposed mechanism that could be partially inhibited through periodontal treatment; however, another plausible mechanism linking CRC and periodontitis is that ectopic oral microbes adhering to the colonic mucosa and disrupting the epithelial barrier, resulting in inflammation, which initiates the inflammatory processes during carcinogenesis [98]. Although in a small and high-risk-of-bias study, periodontal treatment reduced the colonic *F. nucleatum* in patients with colorectal tumours [40]. Given the current evidence, the oral biome is well placed for clinicians as a non-invasive source of biomarkers indicating if further CRC tests are needed.

Cardiovascular disease is the most clinically developed association and the most relevant for clinicians. A meta-analysis of prospective cohorts estimated a 14% higher risk of cardiovascular events, rising to 144% for stroke [99]. Atrial fibrillation has been associated with periodontal plaque accumulation, with the associated acute inflammatory response proposed as the mediating mechanism [100,101]. Coronary heart disease prevalence has been observed as greater in individuals who are *P. gingivalis* seropositive [102]. The evidence for controlling the systemic inflammation pathway through oral–gut microbiome balance is important because of the multiple chronic disease associations, most of which must be managed alongside oral health maintenance. The inflammatory pathway is the most clinically developed of the three pathways and is therefore significant to patients for their understanding of its role in systemic diseases and how to target a healthy oral–gut biome is important. Due to the dysbiosis associated with periodontopathic bacteria, such as *P. gingivalis*, an individual’s cardiovascular risk may rise due to an unstable inflammatory response [103]. A meta-analysis of randomised clinical trials with at least 6 months of follow-up revealed that NSPT can effectively reduce CRP serum levels in an individual with periodontitis to those of healthy individual, or to the same extent as a drug intervention [104]. They estimated a pooled CRP level reduction of 0.69 mg/L after 6 months of treatment. Clinicians should be aware of the biomarkers that indicate that treatment is effective. However, despite evidence for the effectiveness of interventions in lowering inflammatory biomarkers, the evidence on their effect on hard disease endpoints can be unclear owing to an absence of powered randomised controlled trials [105]. Chronic low-grade inflammation from untreated periodontitis may contribute to poor systemic health, resulting in worsening oral health. The above evidence suggests that reducing periodontal inflammation through periodontal therapy has been shown to improve key cardiovascular biomarkers [106].

Recently, research has begun to explore the link between the oral–gut axis and cognitive decline better, with a proposed oral–gut–brain axis connected through the central nervous system and the enteric nervous system [107,108]. It has been proposed that inflammatory mediators released in the oral cavity, and chronic oral infections, which contribute to neuroinflammatory conditions, may contribute to worsening brain function, which could eventually result in neurodegenerative diseases [109,110]. Linking this further to Alzheimer’s disease has been approached tentatively due to confounding risk factors between periodontitis and forms of dementia, with systematic reviews of longitudinal studies presenting conflicting results and characterised by confounding bias [111,112]. Regardless, the plausibility of the mechanism justifies clinicians recording periodontal status in older or cognitively at-risk patients, so the association can be tested prospectively while the confounding is resolved, rather than acting on prematurely.

Periodontal therapy has been shown to positively modulate the gut microbiota in individuals with cirrhosis by lowering systemic inflammation, which in turn could improve quality of life and prevent future severe events such as liver failure and infections [113]. This is important for the clinical context, given the high prevalence of poor periodontal conditions in cirrhotic patients [114]. Moreover, systemic inflammation resulting from oral–gut dysbiosis can result in non-alcoholic fatty liver disease (NAFLD), with clinical and epidemiological evidence supporting an association between periodontitis and NAFLD [115]. This positions periodontal care as a wider concern between dental and medical teams. Clinicians managing cirrhosis or NAFLD can reasonably flag and treat periodontal disease as a modifiable inflammatory contributor, while recognising that the evidence is interventional in cirrhosis but so far is only associative in NAFLD.

Type 2 diabetes is the most actionable association and is a clear example of the pathways combined. Diabetic individuals with periodontal disease exhibit altered omega-3 and omega-6 fatty acid profiles, which shift lipid mediator production to a more pro-inflammatory state [116]. Metabolite-mediated mechanisms, such as SCFAs acting on enteroendocrine cells to release glucagon-like peptide (GLP-1), which enhances insulin secretion, provide an additional mechanism that the inflammatory pathway does not explain [117]. Whether periodontal treatment can significantly influence the metabolic management of T2D is unclear [118]. Dietary improvements, especially increases in fibre, have been shown to improve the metabolic profiles of individuals with periodontal diseases, reflected in decreases in plasma glucose levels, lipoprotein cholesterol and triglycerides [119,120]. A diet optimised for oral health, which includes low refined carbohydrates and high vitamin C and D intake, greatly reduces gingival inflammation and bleeding on probing, most likely due to metabolite-mediated changes, as plaque levels remained unchanged [121]. Dietary changes present clinicians with low cost and beneficial posttreatment options and suggestions to patients, extending care beyond oral health in an accessible manner.

Reduced SCFA production may exacerbate the inflammatory pathway. Elevated TMAO, reduced SCFA production and altered fatty acid profiles are independently associated with diabetic complications, various arterial diseases and cardiovascular diseases [122–126]. We highlight the stacked metabolite-mediated risk that individuals with periodontitis may face through this pathway, and the overlap with the inflammatory pathway demonstrates the clinical relevance of integrated care. Emerging evidence also identifies the oral–gut axis as a potential modifier for controlling diabetes mellitus through glycaemic control and inflammation levels, although heterogeneity in the studies analysed limits conclusions [127]. Studies have shown that even basic home attempts to support the oral microbiome composition can improve the blood pressure profile, and are associated with lowering the risk of heart failure and atrial fibrillation [128,129].

Clinicians supporting patients should highlight the importance of low-cost and low-risk self-management efforts, such as dietary and oral hygiene practices that simultaneously serve the mouth and gut. These factors complement professional periodontal care without imposing financial or clinical burdens on patients. The translocation pathway evidence highlights that oral health is not an isolated factor and that good periodontal health can produce wider systemic effects. In clinical practice, periodontal therapy will target translocation and metabolite-mediated effects simultaneously alongside the inflammatory pathway, which patients and clinicians will welcome given the uncertainty over the role of any one specific mediator.

Although this is a more emerging area, there are important patient consequences that can worsen existing comorbidities; however, the effect in isolation is more speculative than for pathways 1 and 2. The patient consequences for this pathway are therefore broad, and are underlined by efforts that support a healthy oral‒gut axis as a means of controlling systemic disease. These include a healthy diet rich in fibre and omega-3 fatty acids. The same dietary advice that supports periodontal health also supports T2D by supporting glycaemic control and associated cardiovascular risk [79,121,130]. What can be taken from the evidence for this pathway, despite the difficulty in isolating the specific contribution of metabolite signalling against the contributions of the translocation and inflammatory pathways, is that the oral–gut microbiome balance affects the broader health picture. Clinician and patient consequences are less actionable than for other pathways, but we highlight that the benefits of positive behaviours operate through multiple simultaneous mechanisms regardless of which pathway dominates in the individual.

### The oral–gut-medication axis

This section is for hypothesis-generation, and remains speculative based on limited existing evidence that has yet to be supported in controlled studies. Evidence suggests that orally administered drugs can be chemically modified by human gut bacterial strains, with each strain capable of metabolising multiple common drugs [131]. Among 271 commonly administered oral drugs tested against 76 representative human gut bacterial strains, 65% were chemically modified by at least one strain, with each individual strain capable of metabolising between 11 and 95 drugs. Much work has been done to establish the gut microbiome as a significant drug-metabolizing organ, with interpersonal microbiome variation providing an explanation for differences in drug response between individuals, and some drugs that act in other ways and disrupting gut microbiomes [132,133]. The four main mechanisms by which the gut microbiome modifies drug pharmacokinetics are (1) direct transformation by microbial enzymes in the gut lumen; (2) enterohepatic recirculation, where hepatically conjugated metabolites are deconjugated by bacterial *β*-glucuronidases and reabsorbed; (3) modified hepatic metabolism due to gut bile acid and SCFA signalling on CYP450 enzymes; and (4) disturbed luminal conditions due to an unbalanced microbial environment that affects absorption [132,134–136].

Initial mechanistic evidence has been drawn from either *in vitro* or animal models rather than direct human measurement. However, subsequent efforts have been made to establish microbially-modified drug metabolism in humans, and promote the bioaccumulation of drugs within bacterial cells [137]. An established example is sulfasalazine for IBD and rheumatology, which relies on gut bacteria to release sulfapyridine and 5-aminosalicylic acid, the active anti-inflammatory components. A reduced colonic bacterial diversity may yield less of the active drug effect from the same dose [138]. This is an example of microbiome-mediated drug activation, rather than inactivation from gut dysbiosis. What is contested is whether periodontitis-associated changes to the gut microbiome have an extended effect on the microbial functions in the gut that play a role in drug metabolism. Evidence for periodontitis-associated changes in the gut microbial composition extending to functional consequences for drug metabolism is currently unclear. Biological plausibility has been established, and there are clinical trials investigating the drug-gut microbiota dynamics but not the role of periodontal disease in causing gut dysbiosis [133,139,140].

The two parallel literatures – on the role of the gut microbiome in modifying drug metabolism and on the role of oral health in altering the gut microbial composition – have yet to be bridged and integrated. A similar interaction has been researched in other disease contexts, such as that between oral cardiac drugs and the gut microbiota in individuals with cardiovascular diseases [139,141]. This reasoning has not yet been extended to periodontitis. We therefore adopt a deliberate hypothesis-generating approach, drawing on indirect and mechanistic evidence to construct a candidate drug profile and to define testable hypotheses relevant to periodontal care.

Proton-pump inhibitors (PPIs) are used to treat acid-related disorders along the gastrointestinal tract [142]. This class includes omeprazole, esomeprazole, pantoprazole, lansoprazole, rabeprazole, and dexlansoprazole. Large cohort studies have shown that PPIs disrupt up to 20% of taxa in the gut microbiota, and evidence suggests that PPIs lead to an increase in the abundance of oral-originated microbiota taxa in the gut [143–146]. Interestingly, PPIs have been associated with reduced periodontal disease severity in retrospective cohort analyses, potentially through inflammation regulation and effects on the gut microbiome, although the specific causality is difficult to untangle [147,148]. These two observations are not contradictory. Any anti-inflammatory benefit at the periodontal site operates on host inflammation, whereas the compounding effect we propose concerns downstream gut colonisation. PPIs primarily operate through altered luminal conditions, affecting microbial communities and downstream drug absorption, but the relationship also directly intersects with the translocation pathway. By reducing gastric acidity, PPIs reduce a barrier that would typically prevents some oral bacteria from surviving their movement to the gut. One prospective study found that taking PPIs resulted in a 42-fold increase in the prevalence of gut *Streptococcus anginosus*, and that this effect was partially stopped by using mouthwash [149]. In moderate-to-severe periodontitis, the same PPI dose may permit a greater oral bacterial load to reach and colonise the gut, since an identical reduction in the acid barrier acts on a much larger oral pathobiont reservoir. If borne out, this would mean that dental clinicians and physicians may need to interpret a patient’s PPI use differently from that of a periodontally healthy individual and to coordinate medication decisions – a possibility that requires direct study before it can inform practice.

Statins are used to treat hyperlipidaemia by inhibiting 3-hydroxy-3-methylglutaryl coenzyme A reductase (HMG-CoA reductase), an enzyme involved in the synthesis of cholesterol. They also possess antioxidant and antiinflammatory properties, which may help reduce the symptoms of periodontal disease by decreasing IL-1β and increasing IL-10 levels in individuals with periodontitis [150,151]. They have been commonly used as an adjunct to both surgical and non-surgical periodontal therapy, with a significant reduction in periodontal disease associated with their use [152]. Statins interact with the gut microbiome through multiple biological mechanisms. The hepatic UGT enzymes glucuronidate many statins for biliary excretion before bacterial *β*-glucuronidases in the gut deconjugate these metabolites, resulting in an active drug for absorption [134]. Bile acid originating from the gut and SCFA signalling modulate hepatic CYP3A4 expression; consequently, the gut microbial composition indirectly affects how efficiently the liver processes statins [153]. Statin use has also been observed to alter the gut microbiome, causing an increased abundance of Bacteroides and Butyricimonas, which suggests a bidirectional relationship [154]. Furthermore, heterogeneity in the statin response has been shown to be predicted by the composition of an individual’s gut microbiome [155]. Individuals with moderate-to-severe periodontal disease, who exhibit reductions in SCFA-producing taxa and altered bile acid signalling, may plausibly contribute to the inter-individual variability in statin response observed in human cohorts, possibly through modified hepatic CYP3A4 expression. Statins are first-line therapy for cardiovascular risk reduction, which is elevated in periodontally ill individuals because of shared risk factors and potential causation [156]. The consequences of this association are therefore clinically relevant, and future research needs to compare the statin response in those who are periodontally healthy and those who are not.

Metformin lowers blood glucose levels by reducing hepatic glucose production and peripheral insulin resistance, and is therefore frequently used to treat Type 2 diabetes [157]. The majority of metformin’s therapeutic action takes place in the gut, more so than those of PPIs or statins; therefore, the microbiome plays a direct role in how the drug performs. Metformin increases *Akkermansia muciniphila* and SCFA-producing taxa, and modifies bile acid metabolism through the suppression of Bacteroides fragilis-mediated bile salt hydrolase activity [158]. Increased microbial SCFA production stimulates enteroendocrine L-cell GLP-1 release, which enhances glucose-dependent insulin secretion [159]. Metformin used as an adjunct to periodontal therapy has been associated with additional benefits, potentially through increased expression of osteogenic genes and reduced inflammatory burden [160]. Periodontitis-associated reductions in SCFA-producing taxa and altered bile acid signalling may, in principle, reduce the microbiome-mediated effects of metformin. In a large cohort, metformin has also been demonstrated to have a protective effect on the risk of developing gingival and periodontal diseases in diabetic individuals [161]. The bidirectional link between periodontitis and T2D is one of the most clinically established periodontal-systemic links, and with metformin as a first-line T2D medication, this marks the relationship as one of the most clinically actionable [162].

Antibiotics act on the microbial‒immune interface directly rather than being modified by it, and have the potential to disturb the gut‒immune interface even as they benefit the periodontal site. Systemically administered antibiotics reach the gut, where they deplete commensal diversity and SCFA-producing taxa and erode colonisation resistance [163]. SCFAs support barrier integrity and regulatory T-cell differentiation. Their loss shifts gut–immune tone and opens niches for Gram-negative bacteria and taxa originating from the oral cavity [164]. We hypothesise that in moderate-to-severe periodontitis, where the oral pathobiont reservoir is large, the transient collapse of colonisation resistance after an antibiotic course may permit greater and more prolonged ectopic gut colonisation by oral-origin bacteria than the same regimen would in a periodontally healthy individual. This is reinforced by antimicrobial-resistance considerations, since the periodontitis-associated oral and gut microbiome is already enriched for resistance genes, and repeated adjunctive courses may select within a primed reservoir [165].

Notably, attempts to restore the oral–gut community through probiotic coadministration have so far not demonstrated added gut–microbial benefit in a controlled setting, underlining that the disruptive arm of this hypothesis is currently better supported than the restorative one [63].

Whether periodontitis-associated gut dysbiosis alters systemic drug metabolism is directly untested. Genetic evidence does lend indirect support that such a chain is real, with a Mendelian randomisation analysis finding that anti-inflammatory drug-target genes influence oral disease risk, with the effect being partly mediated by the gut microbiota [166]. This demonstrates a drug-target–gut–oral pathway at the genetic level, even if it does not address drug metabolism directly.

Currently, research is being carried out into how the state of an individual’s microbiome will affect the future of precision medicine. The dynamic and personal nature of the gut and oral biomes makes the prediction of drug–microbiota interactions difficult on an individual basis [167]. With the increased interest in precision medicine, it may be beneficial for studies exploring drug efficacy to account for the periodontal disease status of an individual. The unique microbiome signature of each individual will need to be considered in therapeutic regime planning, and whether the effects of periodontal disease-related gut dysbiosis may be considered in the future [168].

## Conclusions

To our knowledge, we provide the first systematic review to map the oral–gut axis across all three pathways in human evidence and extend it to the medication response. We also provide a novel hypothesis-generating section on a potential oral–gut–medication axis, which introduces the idea that oral–gut dysbiosis may alter the efficacy of drugs. This evidence synthesis was composed of clinician relevance and downstream patient consequences in key focus. The current evidence on the oral‒gut axis has several limitations: it is predominantly cross-sectional evidence and therefore lacks the ability to draw causality, prospective studies suffer from short follow-up and inconsistent adjustment in shared confounders, and much of the translocation evidence is built on 16S rRNA sequencing, which by itself cannot distinguish true translocation from shared environmental exposure. In relation to the drugs chosen in the oral–gut medication section, the case we make for proton-pump inhibitors, statins, metformin and antibiotics is one of biological plausibility and research prioritisation, not of demonstrated effect.

## Supplementary Material

Supplementary Material Supplementary material.docx

## Data Availability

Not applicable.
