# Supplementary material for: The oral‒gut axis in periodontal disease: a systematic review of the associated human evidence of its mechanisms and implications for patient care
Source: J Oral Microbiol. 2026 Jul 22;18(1):2705699. doi: 10.1080/20002297.2026.2705699 (PMC13393059; doi:10.1080/20002297.2026.2705699)
Supplement: Supplementary Material — docx [file ZJOM_A_2705699_SM7141.docx]

Supplementary material to: **The Oral-Gut Axis in Periodontal Disease: A systematic review of the associated human evidence of its mechanisms, and implications for patient care**

**Table S1:** Search strings for literature

| **Database** | Periodontitis × oral–gut microbiome / metabolites | Periodontitis × drug metabolism / pharmacokinetics |
| --- | --- | --- |
| **PubMed/MEDLINE** | ("Periodontitis"[Mesh] OR "Periodontal Diseases"[Mesh]  OR "Dental Scaling"[Mesh] OR "Root Planing"[Mesh]  OR "Periodontal Debridement"[Mesh] OR periodontitis[tiab]  OR periodontal[tiab] OR "scaling and root planing"[tiab]  OR "non-surgical periodontal"[tiab]  OR "subgingival instrumentation"[tiab])  AND  ("Gastrointestinal Microbiome"[Mesh] OR "Dysbiosis"[Mesh]  OR "Bacterial Translocation"[Mesh] OR "Endotoxemia"[Mesh]  OR "Fatty Acids, Volatile"[Mesh] OR "Methylamines"[Mesh]  OR "gut microbi*"[tiab] OR "gut microbiome"[tiab]  OR "intestinal microbi*"[tiab] OR "gut dysbiosis"[tiab]  OR "oral-gut"[tiab] OR "oral gut"[tiab] OR "gut-oral"[tiab]  OR "fecal microbi*"[tiab] OR "faecal microbi*"[tiab]  OR "stool microbi*"[tiab] OR "bacterial translocation"[tiab]  OR endotoxemia[tiab] OR endotoxaemia[tiab]  OR "short-chain fatty acid*"[tiab]  OR "short chain fatty acid*"[tiab] OR SCFA[tiab]  OR butyrate[tiab] OR propionate[tiab]  OR trimethylamine[tiab] OR TMAO[tiab]) | ("Periodontitis"[Mesh] OR "Periodontal Diseases"[Mesh]  OR periodontitis[tiab] OR periodontal[tiab]  OR "scaling and root planing"[tiab])  AND  ("Pharmacokinetics"[Mesh] OR "Inactivation, Metabolic"[Mesh]  OR "Proton Pump Inhibitors"[Mesh]  OR "Hydroxymethylglutaryl-CoA Reductase Inhibitors"[Mesh]  OR "Metformin"[Mesh] OR pharmacokinetic*[tiab]  OR "drug metabolism"[tiab] OR "drug response"[tiab]  OR pharmacomicrobiom*[tiab] OR "drug-microbiome"[tiab]  OR "drug microbiome"[tiab] OR "proton pump inhibitor*"[tiab]  OR statin*[tiab] OR metformin[tiab] OR "bile acid*"[tiab]) |
| **Scopus** | TITLE-ABS-KEY(periodont* OR "scaling and root planing"  OR "subgingival instrumentation")  AND TITLE-ABS-KEY("gut microbi*" OR "intestinal microbi*"  OR "gut dysbiosis" OR "oral-gut" OR "oral gut"  OR "fecal microbi*" OR "faecal microbi*"  OR "stool microbi*" OR "bacterial translocation"  OR endotoxemia OR endotoxaemia  OR "short-chain fatty acid*" OR "short chain fatty acid*"  OR SCFA OR butyrate OR propionate  OR trimethylamine OR TMAO) | TITLE-ABS-KEY(periodont* OR "scaling and root planing")  AND TITLE-ABS-KEY(pharmacokinetic* OR "drug metabolism"  OR "drug response" OR pharmacomicrobiom*  OR "drug-microbiome" OR "proton pump inhibitor*"  OR statin* OR metformin OR "bile acid*") |

**Table S2:** Full risk of bias results for studies included.

**A. RoB 2 — randomised trials**

| **First author (year)** | **Rayyan ID** | **D1** | **D2** | **D3** | **D4** | **D5** | **Overall** |
| --- | --- | --- | --- | --- | --- | --- | --- |
| de Oliveira, et al (2021) | 61 | Low | Low | Low | Low | Low | Low |
| Paquette, et al (2000) | 144 | Low | Low | Low | Low | Low | Low |

D1 randomisation process · D2 deviations from intended interventions · D3 missing outcome data · D4 measurement of the outcome · D5 selection of the reported result.

**B. ROBINS-I — non-randomised studies of interventions**

| **First author (year)** | **Rayyan ID** | **D1** | **D2** | **D3** | **D4** | **D5** | **D6** | **D7** | **Overall** |
| --- | --- | --- | --- | --- | --- | --- | --- | --- | --- |
| Mrasori, et al (2021) | 2 | Serious | Moderate | Low | Moderate | Low | Moderate | Low | Serious |
| Guo, et al (2025) | 5 | Serious | Serious | Low | Moderate | Low | Moderate | Low | Serious |
| Nicolaiciuc, et al (2017) | 20 | Serious | Moderate | Moderate | Moderate | Moderate | Low | Moderate | Serious |
| Katagiri, et al (2012) | 21 | Moderate | Moderate | Low | Low | Low | Low | Low | Low |
| Takeuchi-Hatanaka, et al (2025) | 27 | Low | Low | Low | Low | Low | Low | Low | Low |
| Bajaj, et al (2018) | 40 | Serious | Moderate | Low | Moderate | Low | Moderate | Moderate | Serious |
| Kopra, et al (2008) | 73 | Critical | Low | Moderate | Low | Low | Low | Low | Moderate |
| Qiqiang, et al (2012) | 79 | Critical | Low | Low | Moderate | Moderate | Low | Moderate | Low |
| Yoshihara, et al (2021) | 84 | Critical | Serious | Serious | Moderate | Moderate | Low | Moderate | Critical |
| Meng, et al (2013) | 86 | Moderate | Low | Serious | Low | Low | Low | Low | Moderate |
| Velioglu, et al (2025) | 88 | Serious | Moderate | Low | Low | Low | Moderate | Serious | Serious |
| Jacob & Zade (2012) | 94 | Moderate | Moderate | Low | Low | Low | Serious | Serious | Serious |
| Fentoğlu, et al (2010) | 140 | Serious | Low | Low | Moderate | Low | Low | Low | Moderate |
| Fentoğlu, et al (2011) | 141 | Serious | Low | Low | Moderate | Low | Low | Moderate | Serious |
| Baima, et al (2024) | 24 | Moderate | Low | Low | Low | Low | Low | Low | Low |
| Miyauchi, et al (2025) | 121 | Serious | Moderate | Low | Moderate | Moderate | Moderate | Moderate | Serious |
| Citterio, et al (2020) | HS-88 | Serious | Serious | Low | Low | Low | Low | Low | Serious |
| Romano, et al (2019) | HS-89 | Moderate | Low | Low | Low | Low | Low | Low | Moderate |

D1 confounding · D2 selection of participants · D3 classification of interventions · D4 deviations from intended interventions · D5 missing data · D6 measurement of outcomes · D7 selection of the reported result.

**C. Mendelian randomisation appraisal (ROBINS-I framework)**

| **First author (year)** | **Rayyan ID** | **D1** | **D2** | **D3** | **D4** | **D5** | **D6** | **D7** | **Overall** |
| --- | --- | --- | --- | --- | --- | --- | --- | --- | --- |
| Song, et al (2023) | 36 | Moderate | Serious | Low | Low | Low | Moderate | Serious | Moderate |
| Luo, et al (2023) | 53 | Moderate | Serious | Moderate | No information | Low | Low | Serious | Moderate |
| Ye, et al (2023) | 59 | Low | Serious | Low | No information | Low | Moderate | Serious | Moderate |
| Zhao, et al (2025) | 82 | Low | Low | Moderate | No information | Low | Moderate | Serious | Moderate |
| Xu, et al (2024) | 124 | Low | Low | Moderate | No information | Low | Serious | Serious | Moderate |
| Hu, et al (2025) | 118 | Moderate | Serious | Moderate | No information | Low | Moderate | Serious | Moderate |

Domains as ROBINS-I. D4 is commonly “no information” for two-sample MR designs.

**D. Newcastle–Ottawa Scale — cohort studies**

| **First author (year)** | **Rayyan ID** | **S1** | **S2** | **S3** | **S4** | **C1** | **O1** | **O2** | **O3** | **Overall** |
| --- | --- | --- | --- | --- | --- | --- | --- | --- | --- | --- |
| Wu, et al (2022) | 4 | 1 | 0 | 1 | 0 | 0 | 1 | 0 | 0 | Poor (3/9) |
| Zilberstein, et al (2025) | 35 | 1 | 1 | 1 | 0 | 1 | 1 | 0 | 0 | Fair (5/9) |
| Pussinen, et al (2007) | 67 | 1 | 1 | 1 | 1 | 2 | 1 | 1 | 1 | Good (9/9) |
| Liljestrand, et al (2017) | 100 | 1 | 1 | 1 | 0 | 2 | 1 | 0 | 0 | Fair (6/9) |
| Ye, et al (2023) | 101 | 1 | 1 | 1 | 0 | 2 | 1 | 0 | 0 | Fair (6/9) |
| Imai, et al (2021) | 102 | 1 | 1 | 1 | 1 | 1 | 1 | 1 | 1 | Good (8/9) |
| Yama, et al (2023) | 111 | 1 | 1 | 1 | 1 | 1 | 1 | 1 | 0 | Good (7/9) |
| Miyauchi, et al (2025) | 122 | 1 | 1 | 1 | 0 | 2 | 1 | 0 | 0 | Fair (6/9) |
| Xu, et al (2026) | 129 | 1 | 1 | 1 | 0 | 2 | 1 | 0 | 0 | Fair (6/9) |
| Conway, et al (2008) | 142 | 0 | 1 | 1 | 0 | 0 | 1 | 1 | 1 | Fair (5/9) |
| Martu, et al (2026) | 145 | 1 | 1 | 1 | 0 | 2 | 1 | 0 | 0 | Fair (6/9) |

Cells show stars awarded (0/1; comparability 0–2). Overall rating derived from total: Good ≥7, Fair 5–6, Poor ≤4.

**E. Newcastle–Ottawa Scale — case–control studies**

| **First author (year)** | **Rayyan ID** | **S1** | **S2** | **S3** | **S4** | **C1** | **E1** | **E2** | **E3** | **Overall** |
| --- | --- | --- | --- | --- | --- | --- | --- | --- | --- | --- |
| Wang, et al (2026) | 54 | 1 | 1 | 1 | 1 | 2 | 1 | 1 | 0 | Good (8/9) |
| Palm, et al (2014) | 106 | 1 | 1 | 1 | 1 | 1 | 1 | 1 | 0 | Good (7/9) |

Cells show stars awarded (0/1; comparability 0–2). Overall rating derived from total: Good ≥7, Fair 5–6, Poor ≤4.

**F. JBI critical appraisal — analytical cross-sectional studies**

| **First author (year)** | **Rayyan ID** | **Q1** | **Q2** | **Q3** | **Q4** | **Q5** | **Q6** | **Q7** | **Q8** | **Overall** |
| --- | --- | --- | --- | --- | --- | --- | --- | --- | --- | --- |
| Al-kuraishy, et al (2019) | 8 | Yes | Yes | Unclear | Yes | Yes | No | Yes | Yes | Include |
| Fentoğlu, et al (2010) | 11 | Yes | Yes | Yes | Yes | Yes | Yes | Yes | Yes | Include |
| Elimelech, et al (2015) | 18 | Yes | Yes | Yes | Yes | Yes | Yes | Yes | Yes | Include |
| Bao, et al (2022) | 23 | Yes | Yes | Yes | Yes | Yes | No | Yes | Yes | Include |
| Britos, et al (2025) | 26 | Yes | Yes | Yes | Yes | Yes | Yes | Yes | Yes | Include |
| Paz, et al (2025) | 28 | Yes | Yes | Yes | Yes | Yes | No | Yes | Yes | Include |
| Baêta Lourenço, et al (2022) | 30 | Yes | Yes | Yes | Yes | Yes | Yes | Yes | Yes | Include |
| Wu, et al (2024) | 31 | Yes | Yes | Yes | Yes | Yes | Yes | Yes | Yes | Include |
| Ribeiro, et al (2026) | 33 | Yes | Yes | Yes | Yes | Yes | Yes | Yes | Yes | Include |
| Serdar, et al (2025) | 48 | Yes | Yes | Yes | Yes | Yes | No | Yes | Yes | Include |
| Bal, et al (2025) | 50 | Yes | Yes | No | Yes | Yes | Yes | Yes | Yes | Include |
| Zhou, et al (2021) | 60 | Yes | Yes | Yes | Yes | Yes | Yes | Yes | Yes | Include |
| Li, et al (2020) | 65 | Yes | Yes | Yes | Yes | Yes | Yes | Yes | Yes | Include |
| Yang, et al (2020) | 74 | Yes | Yes | No | Yes | Yes | Yes | Yes | No | Include |
| Ding, et al (2023) | 81 | Yes | Yes | Yes | Yes | No | No | Yes | Yes | Include |
| Thouvenot, et al (2026) | 91 | Yes | Yes | Yes | Yes | Yes | No | Yes | Yes | Include |
| Buetas, et al (2024) | 97 | Yes | Yes | Yes | Yes | Yes | Yes | Yes | Yes | Include |
| Rashid, et al (2025) | 98 | Yes | Yes | Yes | Yes | No | No | No | No | Include |
| Hayashi, et al (1999) | 105 | Yes | Yes | Yes | Yes | Yes | Yes | Yes | Yes | Include |
| Amado, et al (2020) | 109 | Yes | Yes | Yes | Yes | Yes | Yes | Yes | Yes | Include |
| Franco-Duarte, et al (2025) | 117 | Yes | Yes | Yes | Yes | Yes | Unclear | Unclear | Yes | Include |
| Nishimoto, et al (2023) | 119 | Yes | Yes | Yes | Yes | Yes | Unclear | Unclear | Yes | Include |
| Kamer, et al (2024) | 120 | Yes | Yes | Yes | Yes | Yes | Yes | Yes | Yes | Include |
| Lourenςo, et al (2018) | 123 | Yes | Yes | Yes | Yes | Yes | No | Yes | Yes | Include |
| Yay, et al (2024) | 126 | Yes | Yes | Yes | Yes | Yes | No | Yes | Yes | Include |
| Kawamoto, et al (2021) | 127 | Yes | Yes | Yes | Yes | Yes | Yes | Yes | Yes | Include |
| Na, et al (2021) | 130 | Yes | Yes | Unclear | Yes | No | No | Yes | No | Include |
| Zhang, et al (2021) | 132 | Yes | Yes | Yes | Yes | Unclear | Unclear | Yes | Yes | Include |
| Fentoğlu, et al (2011) | 136 | Yes | Yes | Unclear | Yes | Yes | Yes | Yes | Yes | Include |
| Ari, et al (2016) | 137 | Yes | Yes | Unclear | Yes | Unclear | No | Yes | No | Include |
| Suresh, et al (2013) | 138 | Yes | Unclear | Yes | Yes | Unclear | Yes | Yes | No | Include |
| Tomás, et al (2017) | 139 | Yes | Yes | Yes | Yes | Unclear | Yes | Yes | Yes | Include |
| Miyauchi, et al (2025) | 121 | Yes | Unclear | Yes | Yes | Unclear | No | Yes | Yes | Include |
| Biama, et al (2024) | 24 | Yes | Yes | Yes | Yes | Yes | Yes | Yes | Yes | Include |
| Baima, et al (2025) | HS-39 | Yes | Yes | Yes | Yes | Yes | Unclear | Yes | Yes | Include |
| Liebsh, et al (2019) | HS-85 | Yes | Yes | Yes | Yes | Yes | Yes | Yes | Yes | Include |
| Kim, et al (2021) | HS-86 | Yes | Yes | Unclear | Yes | Yes | Unclear | Yes | Yes | Include |
| Barnes, et al (2014) | HS-93 | Yes | Yes | Unclear | Yes | Yes | No | Yes | Yes | Include |

Q1 inclusion criteria · Q2 subjects & setting · Q3 exposure measured validly · Q4 objective standard criteria · Q5 confounders identified · Q6 confounder strategy · Q7 outcomes measured validly · Q8 appropriate statistics. Overall = inclusion decision.

**G. JBI critical appraisal — quasi-experimental studies**

| **First author (year)** | **Rayyan ID** | **Q1** | **Q2** | **Q3** | **Q4** | **Q5** | **Q6** | **Q7** | **Q8** | **Q9** | **Overall** |
| --- | --- | --- | --- | --- | --- | --- | --- | --- | --- | --- | --- |
| Fentoğlu, et al (2015 | 7 | Yes | Yes | Yes | No | No | Unclear | Yes | Yes | Yes | 6/9 |

Q1–Q9 JBI quasi-experimental items. Overall = summed score /9.
